# Atomic pair distribution functions from textured polycrystalline samples: fundamentals

**DOI:** 10.1107/S2053273325007387

**Published:** 2025-09-18

**Authors:** Zizhou Gong, Songsheng Tao, Simon J. L. Billinge

**Affiliations:** a Department of Physics Columbia University,New York NY 10027 USA; b Department of Applied Physics and Applied Mathematics Fu Foundation School of Engineering and Applied Sciences Columbia University,New York NY 10027 USA; Institute of Crystallography - CNR, Bari, Italy

**Keywords:** pair distribution function, PDF, crystallographic texture, preferred orientation, total scattering, nanostructure determination, orientation distribution function, ODF, bond orientation distribution function, BODF

## Abstract

Equations for the reduced structure function and atomic pair distribution function (PDF) of a textured polycrystalline sample are formulated in terms of the orientational distribution function (ODF) and the structure function from a single crystallite. This ODF is sensitive to orientational distributions of inter­atomic vectors and to differentiate it from the crystallographic case we call it the bond orientational distribution function (BODF). This BODF may be obtained from experimental data when the structure of the reference crystallite is known. It can be applied to nanocrystalline and amorphous samples going beyond the information present in a conventional crystallographic texture study.

## Abstract 2

The basic equations for the atomic pair distribution function of a sample that has a crystallographic texture (preferred orientation) are derived.

## 1. Introduction

The atomic pair distribution function (PDF) analysis of X-ray, neutron and electron diffraction data is becoming a widely used method for studying the structure of nanomaterials (Laveda *et al.*, 2017 ▸; Page *et al.*, 2011 ▸; Stein *et al.*, 2017 ▸). Because the methodology does not presume periodic long-range order of the underlying lattice, as is the case in traditional Bragg crystallography, this approach may be extended to nanostructured and disordered systems. The most commonly applied PDF method starts from the ideal powder approximation, resulting in the 1D PDF, which is simply a histogram of the interatomic distance distributions in the sample (Duxbury *et al.*, 2016 ▸; Billinge *et al.*, 2016 ▸). This approximation is a good one for the vast majority of nanocrystalline samples, in part because for nanomaterials the small grain size results in rather good powder averaging. However, with the recent development of thin-film PDF methods (Jensen *et al.*, 2015 ▸), and the desire to measure nanomaterials in different geometries, the specter of preferred orientation and crystallographic texture is becoming an issue even for nano-sized grains. There are also efforts to actually engineer orientationally ordered nanoparticle assemblies (Xu *et al.*, 2020 ▸).

There is no reason, in principle, why the PDF equations cannot be extended to the case of a textured polycrystalline sample. Here we develop the basic equations for the total scattering structure function and the atomic PDF of a textured polycrystalline sample. These equations were originally posted to a preprint server (Gong & Billinge, 2018 ▸) but now appear here after full peer review and with a slightly expanded demonstration section. The equations have been successfully applied in a more extensive experimental setting (Harouna-Mayer *et al.*, 2022 ▸). We also note the related development of equations for correcting Debye scattering equations for crystallographic texture (Cervellino & Frison, 2020 ▸) as well as attempts at obtaining this information with higher-order correlation functions (Binns *et al.*, 2022 ▸).

## 2. Definition of the polycrystalline structure function, *S_p_*(**Q**), and polycrystalline PDF *G_p_*(**r**)

As a start, we write down the full 3D structure function (Egami & Billinge, 2012 ▸), 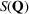, which may be obtained from measured scattering intensities, 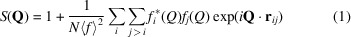

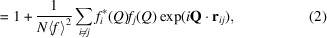
where 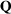 is the scattering vector, 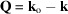, where 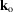 and 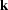 are the wavevectors of the incident and scattered waves, respectively, and 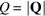 is the magnitude of the scattering vector. *N* is the number of atoms in the (illuminated part of the) sample and 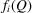 is the atomic form factor of the *i*th atom. 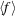 is the sample average structure, given by 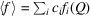. The sums over *i* and *j* run over every atom in the sample in a way that avoids double-counting, where equation (2) serves to define 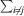. Finally, 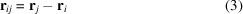
is the vector joining atom *i* and atom *j*, where 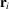 is the vector from the origin of the sample reference frame to the *i*th atom.

The sample reference frame is a coordinate system on the sample. This can be any valid coordinate system but, in practice, it is chosen by the experimenter in some way that reflects the geometry of the situation: for example, placing the *z* direction along the axis of a wire and *x* and *y* in convenient perpendicular directions.

The function 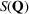 defined in equations (1) and (2) is proportional to the scattered intensity from the sample at every point 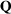 in reciprocal space. In practice, it is measured by reorienting the sample with respect to the incident beam in such a way as to capture the sample scattering at every point in 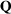. Each measurement is mapped back to the sample reference frame to yield 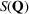 (Estermann & Steurer, 1998 ▸).

For an isotropic powder, the sample does not have to be reoriented at all to obtain complete information (though the sample is often spun to improve powder statistics). In general, to sample the specimen scattering for the full reciprocal space in an experiment with a large area 2D detector it is possible simply to rotate the sample around one axis, or two non-collinear axes, that are perpendicular to the incident beam direction. Two axes may be required if there is a missing wedge due to an inability to rotate the sample fully around one axis for some reason. For brevity, we will refer to this as the orthogonal axes rotation (OAR) approach.

If the sample has intermediate symmetries, for example a fiber symmetry, the sample reorientation scheme can be modified to take advantage of this, though the ease of application of the OAR method means that, in practice, it is often the method of choice. We note that these are standard approaches for measuring texture in crystalline samples; however, in the current context it is important to collect with accuracy all the diffuse scattering between the Bragg peaks which is not considered in conventional texture experiments (which just measure the Bragg peak intensities).

To obtain 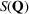 from the measured intensities we make corrections for experimental artifacts, such as various sources of parasitic scattering and multiplicative aberrations such as polarization, absorption and so on. We also normalize by the incident flux (Egami & Billinge, 2012 ▸).

In this way, the structure function 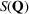 may be obtained from any sample with any degree of anisotropy. For example, when the sample is a single crystal, this function constitutes the 3D crystallographic structure function which may be Fourier transformed to obtain the 3D PDF (Egami & Billinge, 2012 ▸). The 3D PDF is emerging as a powerful approach for studying diffuse scattering and defects in single crystals (Weber & Simonov, 2012 ▸; Egami & Billinge, 2003 ▸). It is given by 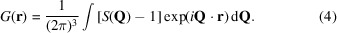


Here we are interested in the particular case where we have a sample that is polycrystalline but has some texture or preferred orientations of crystallites. The crystallites could be bulk-sized crystals, which is the familiar and widely studied case of a textured polycrystalline sample (Bunge, 1982 ▸). However, as with all PDF studies, we do not presume long-range order, and the crystallites could be nano-sized in principle, and, as we see later, even amorphous. Collecting the entire reciprocal space is becoming highly feasible these days with the use of high-energy X-rays at synchrotron sources coupled with large area photon-counting detectors (Schaub *et al.*, 2007 ▸; Weber & Simonov, 2012 ▸; Osborn & Welberry, 1990 ▸; Welberry *et al.*, 1998 ▸; Welberry & Proffen, 1998 ▸; Proffen & Welberry, 1997 ▸) and with neutron diffraction instruments designed for this purpose (Rosenkranz & Osborn, 2008 ▸; Keen *et al.*, 2006 ▸; Frost *et al.*, 2010 ▸; Tamura *et al.*, 2012 ▸).

We seek to understand how scattered intensities from a textured sample may be propagated through the Fourier transform to obtain a scientifically relevant real-space pair correlation function, and, in principle, how to model that function to obtain information about the texture.

To explore this, we first consider a sample that is an isotropic powder with a large number of grains equally sampling all orientations (a good powder average). In this case, we can average azimuthally to obtain a 1D function, 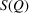. This results in the regular 1D PDF, 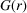, when Fourier transformed.

Let us now consider the simplest textured case, where the sample is made up of two identical crystallites of the same material that are misoriented with respect to each other, and far enough apart that they are both in the incident beam but beyond the coherence volume of the beam. In other words, we assume that scattering from each crystallite is incoherent and the total observed scattering is just the linear superposition of the scattering from each crystallite (we will assume incoherent scattering between crystallites from now on). We define 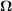 as the three-vector that contains the Euler angles defining the relative orientation of one crystallite with the other one. For convenience, and without loss of generality, we assume the sample reference frame is the reference frame of one of the crystallites, which we call the reference crystallite. If we measured either one of the crystallites as an individual single crystal using the OAR approach, we would get the same single-crystal structure function. However, the measurement is carried out in such a way that the signals from the two crystallites are superposed on the detector. The crystallite structure function can be determined if we are able to separate the superposed signals from each crystallite. For crystalline materials this separation is straightforward; this approach is called polycrystallography and has been developed to a high level (Poulsen, 2004 ▸).

This reasoning is readily extended to the case of *M* separable diffraction patterns from *M* crystallites. In this case, as before, a unique reference frame is defined on a reference crystallite on the sample, which we call the sample reference frame, and we define 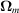 as being the Euler angles that give the orientation of the *m*th crystallite with respect to this reference frame. If 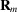 is the rotation matrix that rotates the sample reference frame onto the *m*th crystallite reference frame, we have the following relation:
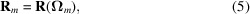

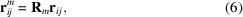
where 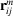 refers to the 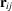 interatomic vector of the reference crystallite, but in the *m*th crystallite at orientation 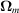. We can thus write the polycrystalline sample structure function 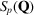 as 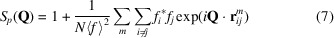

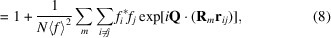
where for notational simplicity we have dropped the explicit *Q*-dependence of the atomic form factors. The double sum over *i* and *j* is now a sum over the interatomic vectors between just the atoms in the reference crystallite and the sum over *m* is a sum over all the (assumed to be) identical but misoriented crystallites. In equation (8) the signal for the polycrystalline sample is built up by rotating the reference crystallite to the orientation of each crystallite in the sample.

We now turn to a polycrystalline sample with a large number of crystallites where the scattering from the individual crystallites is no longer separable, but the sample is still not isotropic: a textured powder. The patterns from the individual grains strongly overlap and multiple crystallites contribute to each region (voxel) of reciprocal space defined by the 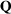 resolution of our measurement. In this case, we would like to convert equation (8) to a continuous function. We define a volume element 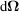 in the Euler angle space that runs from 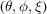 to 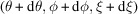. We can then define the number of crystallites in the beam that have an orientation such that their Euler angles place them in that volume element of angle space as 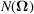.

Now, returning to equation (8), we would like to rewrite this equation in terms of a sum over all orientation directions rather than a sum over *m*. Denoting the total number of crystallites within the sample as 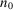, the total number of atoms in the sample, *N*, is then given by 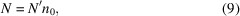
where 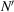 is the number of atoms in the reference crystallite. The sum over *m* then becomes 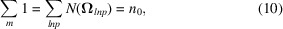
where *lnp* labels voxels in the orientation space and the sum runs over all voxels in the orientation space. Furthermore, since the crystallite with the same orientation 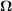 gives the same contribution to 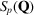, we can rewrite 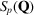 as the summation over different crystallite orientations, weighted by the number of crystallites with that orientation: 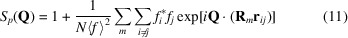

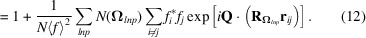
For crystallites oriented quasi-continuously in every orientation, we rewrite this equation with an integral, using 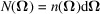, 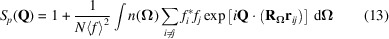

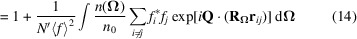

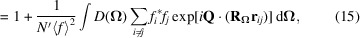
where we introduce the orientation distribution function (ODF), 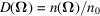. This function serves the purpose of the ODF in crystallographic texture, but rather than just encoding the orientational distribution of crystallites, it encodes the orientational distribution of interatomic vectors. For brevity, and to emphasize this fact, we call it the bond orientational distribution function (BODF). In practice, 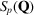 can be evaluated by exchanging the order of the summation over *i* and *j* with the integration over 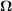 and evaluating the integral involving the BODF and the complex exponential factor.

Here in equation (15) the function 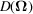 has the meaning of the fraction of the crystallites with orientation 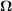 among all crystallites in the sample. It is a sample-dependent property, not depending explicitly on sample orientation, and is expressed in the sample reference frame. Since by definition the BODF is a probability density, it has the normalization property:
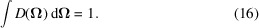
In the special case that we have considered here, the sample is assumed to consist of many identical crystallites that all have the same structure function, 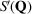, as the reference crystallite but are oriented with respect to that crystallite by 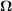. To capture this, we introduce a generalized structure function, for the ‘misoriented’ crystallites, 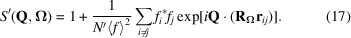
We can then rewrite the polycrystalline sample structure function in terms of 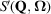, taking advantage of the normalization property of the BODF in equation (16). First, we change the order of integration and summing, 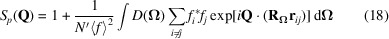

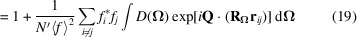

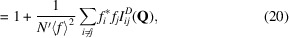
which serves to define the integral 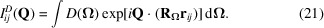
Now, taking advantage of the normalization property of our BODF we can write 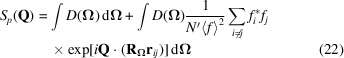

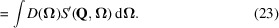
Equation (23) expresses the structure function of the sample as an orientational distribution weighted arithmetic average of the structure function of the reference crystallite.

We note that equation (23) for the case of discrete and separable crystallites may also be rewritten in this way as 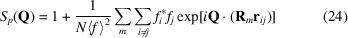

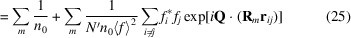

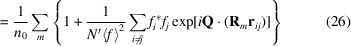

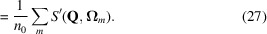
Equations (27) and (23) hold for the approximation that the sample is made up of multiple identical crystallites, or nanoparticles, that have different orientations.

We now consider how this propagates through the Fourier transform to yield a textured polycrystalline pair correlation function, 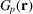, 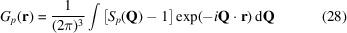

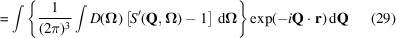

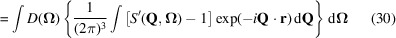

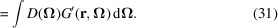
Following the definition of PDF in equation (4), 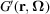 is the 3D PDF of the reference crystallite but with orientation 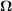, with respect to the sample reference frame, expressed as 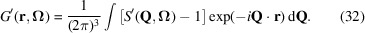


These equations serve to define the real- and reciprocal-space representations of textured polycrystalline samples. In general, 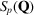 may be measured in the same way as we measure the 3D PDF of a single crystal, for example using the OAR method with X-rays or in a neutron single-crystal experiment. If, as is often the case, we know 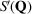, the structure function of the reference crystallite, we can build up the polycrystalline intensity at each 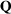 by rotating 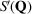 to all angles and adding the contribution. We note that the derivation did not assume crystallinity of the sample, and so it is equally applicable to polycrystalline textured nanoparticle samples and non-isotropic amorphous samples, provided that in these samples the approximation that the local clusters are all equivalent to each other apart from their orientation holds. A similar approach could be carried out directly in real space to determine 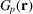. If it is desired to determine the BODF, this approach may be implemented in a regression loop.

## 3. Testing the approach

In this section we demonstrate that this approach may be used to recover the BODF of a simulated textured nickel powder. We have written a program in the Python software language to compute the 3D PDF of a crystalline material; then, given a BODF function, it will compute the 3D PDF that would be obtained from a powder made up of crystallites of the single crystal with the texture given by the BODF. This is the function that would be obtained by measuring the total scattering signal from a textured sample at every orientation and carrying out a 3D Fourier transform, *i.e.* by treating a textured polycrystalline sample as a single crystal and performing a 3D PDF experiment on it. We have further written a program that will carry out regression to obtain a best-fit BODF function, given a textured 3D PDF and a known single-crystal structure. As a proof-of-principle demonstration, we consider a sample of face-centered cubic (f.c.c.) nickel with a fiber texture whose BODF may be expressed as an expansion in spherical harmonics. The allowed non-zero spherical harmonic coefficients are constrained by the cylindrical symmetry of the fiber texture and the cubic symmetry of the crystal. For our testing we have chosen the situation with the sample *z* axis on the axis of the fiber texture and parallel to the [001] direction of the Ni lattice which allows us to write the ODF in the simple form (Bunge, 1982 ▸) 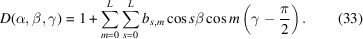
Here *m* and *s* are integer indices that must satisfy 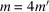 and 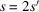 (*i.e.s* is even and *m*/2 is even) for our symmetries and 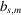 is the coefficient of the *s*,*m*-th term in the expansion. α, β and γ are the Euler angles corresponding to rotations around the sample *z*, *x* and *z* axes (ZXZ convention), respectively. For a better demonstration, a simple BODF is used in the computation of examples below where only 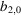 is set to be non-zero so that 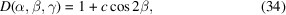
with 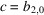. Note that in this setting a uniform distribution (non-textured powder) will be the case where *c* = 0.

The 3D PDF of the f.c.c. crystal is computed in the normal way as 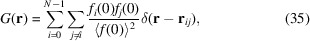
which at orientation 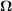 gives us 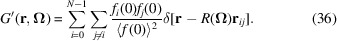
This can be substituted into equation (31) to get the textured polycrystalline pair correlation function. For the purposes of the computer program, this needs to be expressed as a sum on a discrete numerical grid rather than an integral, giving 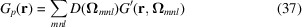

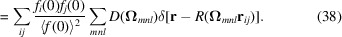
The results of testing the program on an f.c.c. sample (nickel was used) for the weak fiber texture described above are discussed below.

The concept of a pole figure in reciprocal-space mapping of texture is of real practical value because it is possible to consider the powder Bragg peak from a single set of lattice planes and map its intensity as a function of sample orientation in an experiment. In other words, the pole figure is a straightforwardly determinable experimental observable. The full ODF can then be determined from a rather small number of independent pole figures (Bunge, 1982 ▸).

**Figure 1:**
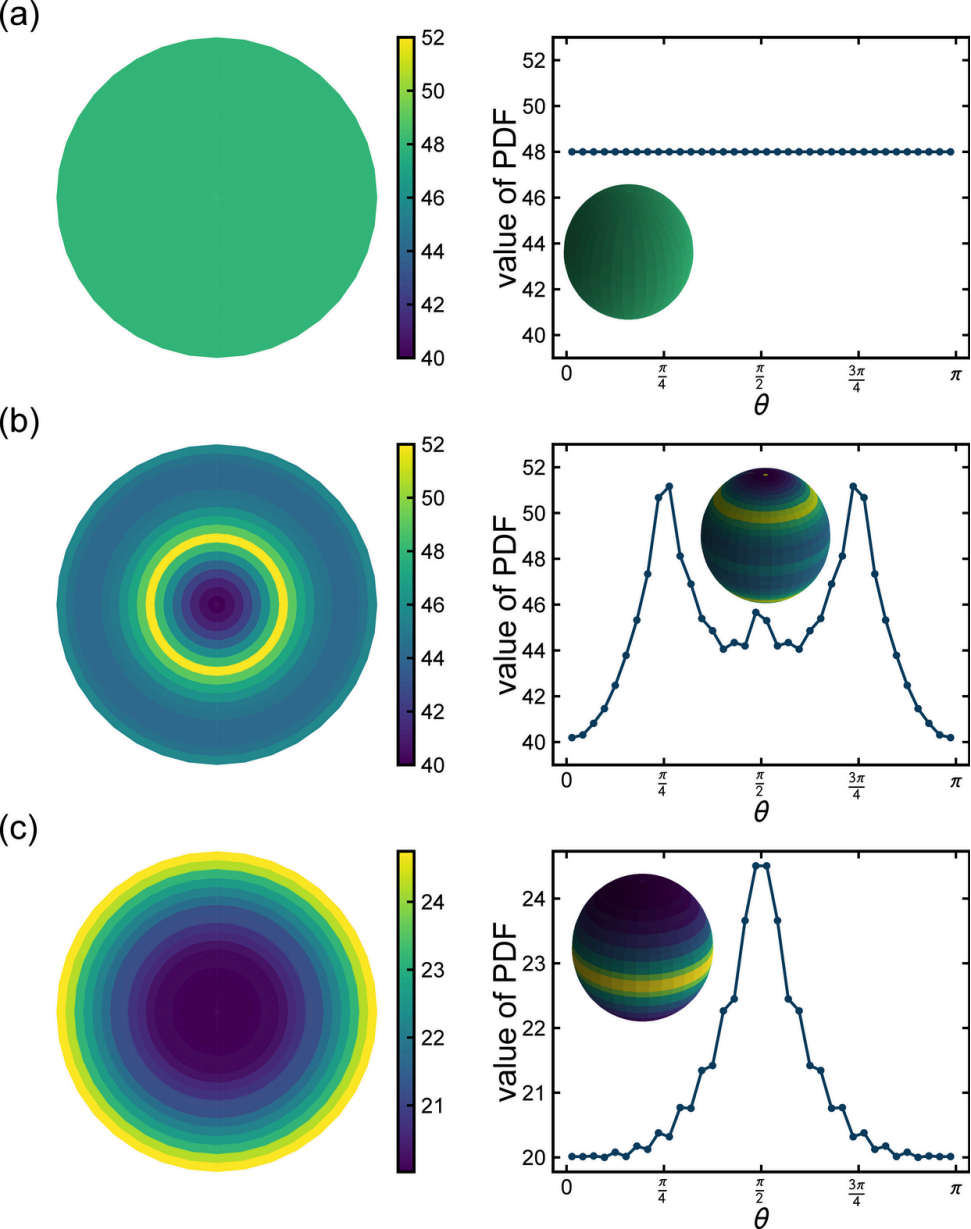
(insets) Plots of the normalized textured polycrystalline PDF, 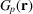 (NTP-PDF), from a nickel f.c.c. sample with a weak fiber texture along the [001] crystallographic direction. See text for details. In (*a*), (*b*) and (*c*) the inset shows a 3D rendering of the NTP-PDF at a fixed *r* value. The left column shows the stereographic projection of the inset. The right column shows a 1D plot of the value in each pixel along a great circle from the north pole to the south pole, as a function of polar angle, θ. (*a*) Case for *r* = 2.49 Å which captures the nearest-neighbor peak for the isotropic sample. (*b*) Same value of *r*, but for the sample with the weak fiber texture given by equation (34) with *c* = 0.5. (*c*) Same texture as (*b*) but for the annulus at *r* = 3.52 Å corresponding to the second-neighbor peak in the PDF. The color scale is the value of the PDF in each pixel.

The real-space pole figure may not be of such practical use since it is not a directly measurable quantity. However, it is a conceptually helpful mechanism for analyzing the results of the calculation, or a 3D PDF measurement of a polycrystalline sample. The real-space inverse pole figure (which projects the bond distributions with respect to a particular sample vector) will be of particular interest. It is obtained from the textured polycrystalline PDF, 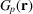, by considering a thin annulus at some fixed value of interatomic distance 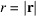 that contains a set of symmetry-equivalent interatomic vectors. For an isotropic sample the BODF is uniform and we will recover a 3D PDF that consists of concentric spherical annuli of uniform intensity centered on the origin at radii corresponding to the values of *r* where 1D PDF peaks occur. We have verified that our program returns this, as shown in the inset to Fig. 1 ▸(*a*), which shows the spherical annulus for the nearest-neighbor peak in Ni at *r* = 2.49 Å. The quantity plotted in the figure is proportional to 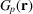, but is not exactly 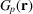. In detail, it is the number of vectors that terminate in that volume element after applying the BODF to a single unit cell of the material. For the isotropic case, as here, this would simply return a value in each pixel that is the multiplicity of the vector being plotted (assuming only one symmetry-equivalent interatomic vector has length falling in this annulus). The calculation was done on the conventional, non-primitive, f.c.c. unit cell containing four Ni atoms, and so the multiplicity for the nearest-neighbor peak plotted is 48. Each volume element should therefore have a value 48 for this isotropic case, as is seen in Fig. 1 ▸(*a*). In the right panel we also show a 1D plot of the values along a longitudinal great line from the north pole to the south pole and obtain again 48 at all points on the surface.

To strengthen the connection to the pole figure of reciprocal-space texture analysis, we have also represented the plots of 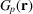 at fixed *r* values in Fig. 1 ▸ as stereographic projections, a common way of representing pole figures, as shown in the left column of the figure. However, the analogy should not be taken too far as these are plots of the density of interatomic vectors versus rotation angle, and not precisely the density of direct-space lattice vectors versus rotation angle. This real-space pole figure may be determined experimentally by measuring the full 3D PDF of the sample and Fourier transforming it, at which point it is possible to determine the full BODF and multiple real-space pole figures. Thus, other than as a useful way of representing the texture visually, the pole figure may not be of quite such central importance in PDF texture experiments as it is in studies of textured crystals.

For the case of the textured sample the spherical annuli occur at the same radial distances, but the intensity varies in orientation, as shown in the insets to Figs. 1 ▸(*b*) and 1 ▸(*c*).

Since the fiber axis is along the sample *z* axis, the BODF is independent of the rotation angle around the polar axis in the 3D PDF plots and 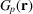 of our textured Ni should be invariant in latitude, as we find in the simulated PDFs.

Having created a program that can compute the 3D PDF of a textured polycrystalline sample, 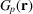, we can compute this and use it as a simulated measured 3D PDF and see whether it is possible to carry out a regression experiment to recover the BODF of a sample from a measured 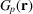 in principle. As a proof of principle, we have implemented this regression program for the fiber texture case and successfully determined the correct value for *c* = 0.5 from a starting point of a uniform BODF (*c* = 0). The regression is linear in the coefficients of the spherical harmonics and as long as a spherical harmonics expansion can give a good approximation to the sample texture, we expect that this general approach will work well even for more complex symmetries and for experimental data. A graphical user interface program (*diffpy.fourigui*) implementing this can be found at https://github.com/diffpy/diffpy.fourigui and was described by Harouna-Mayer *et al.* (2022 ▸).
